# Performance and healthcare analysis in elite sports teams using artificial intelligence: a scoping review

**DOI:** 10.3389/fspor.2024.1383723

**Published:** 2024-04-18

**Authors:** A. A. Munoz-Macho, M. J. Domínguez-Morales, J. L. Sevillano-Ramos

**Affiliations:** ^1^ Computer Architecture and Technology Department, University of Seville, Seville, Spain; ^2^ Performance and Medical Department, Real Club Deportivo Mallorca SAD, Palma, Spain

**Keywords:** artificial intelligence, sports teams, complex systems, sports performance, injury prevention, healthcare

## Abstract

**Introduction:**

In competitive sports, teams are increasingly relying on advanced systems for improved performance and results. This study reviews the literature on the role of artificial intelligence (AI) in managing these complexities and encouraging a system thinking shift. It found various AI applications, including performance enhancement, healthcare, technical and tactical support, talent identification, game prediction, business growth, and AI testing innovations. The main goal of the study was to assess research supporting performance and healthcare.

**Methods:**

Systematic searches were conducted on databases such as Pubmed, Web of Sciences, and Scopus to find articles using AI to understand or improve sports team performance. Thirty-two studies were selected for review.

**Results:**

The analysis shows that, of the thirty-two articles reviewed, fifteen focused on performance and seventeen on healthcare. Football (Soccer) was the most researched sport, making up 67% of studies. The revised studies comprised 2,823 professional athletes, with a gender split of 65.36% male and 34.64% female. Identified AI and non-AI methods mainly included Tree-based techniques (36%), Ada/XGBoost (19%), Neural Networks (9%), K-Nearest Neighbours (9%), Classical Regression Techniques (9%), and Support Vector Machines (6%).

**Conclusions:**

This study highlights the increasing use of AI in managing sports-related healthcare and performance complexities. These findings aim to assist researchers, practitioners, and policymakers in developing practical applications and exploring future complex systems dynamics.

## Introduction

The inception of artificial intelligence (AI) can be traced back to the mid-20th century, marked by the aspiration to create machines capable of emulating human intelligence (1). Over the decades, advancements in computational power and algorithmic sophistication, as well as the availability of large data sets, have propelled AI from theoretical frameworks to practical applications in diverse fields (2).

In the context of sports, one of the first applications of AI was biomechanics modelling (3) and its integration began to gain traction in the early 21st century, evolving from basic statistical analyses to complex predictive modelling and real-time decision-making systems. This progression has enabled sports scientists and professionals to harness AI for performance analysis, biomechanics, sports technique, strategic planning, performance analysis, and improving competitive edge in team sports (4).

During these last years, there has been a great evolution and applications of AI and ML in sports, and some authors have defined the key challenges for AI usage in elite sports, including correct data collection, the process of connecting AI and elite sports communities, the need to keep control in the hands of practitioners, maintaining the explainability of AI results, developing robust predictive models, and closing the loop defined as the need to provide feedback to the AI system to develop quality and self-adaptation (5).

When considering the implementation of AI in elite sports teams, it is critical to successfully address the obstacles associated with human-centered activities such as privacy protection, ethical design, adherence to human principles, governance and oversight, and the preservation of human cognitive abilities (6).

### Elite sports teams, performance, and healthcare

Defining elite sports teams is not a trivial task. Some authors claim that it is a concept that undermines the external validity of high-performance requirements (7). This study focusses on clarifying and proposing some indicators to develop a correct definition development, including: age, competition level, league status, gender, international ranking, nationality, province/state, sport and success/achievements.

Performance in elite sports teams is a wide concept because many different aspects should be considered, such as technical-tactical and those related to physical factors (8). From the perspective of this research, the strength and conditioning of athletes have been considered, as well as physical and physiological aspects. Some authors have previously developed the physical and physiological profiles in team sports such as male football (9) or female football players (10).

Regarding physical training, key performance goals could be considered such as the conceptualization of load (11), the effects of the accumulated training and match load (12) and the ways to monitor it (13). The integration of new devices such as global positioning systems (GPS) during practise and competition has been very important (14),

Key identifying performance markers is challenging, but we can quantify repeated sprint ability (15) injury impact on the team (16) and availability (17). Also crucial is the effect of the competition's arrangement with concatenated matches (18) and fixtures congestion (19). As a performance tool, AI could help to develop recommendations to determine player wellness, trip planning, and sleep management (20).

Healthcare may be conceptualised as a system that combines health management and performance coaching to provide comprehensive care for elite athletes and, eventually, elite sports teams, as opposed to merely ensuring the absence of disease and injury (21). Thus, psychological concerns are an additional component of this approach (22).

### Complex systems and artificial intelligence approach

Elite sports teams are complex systems with interconnected parts that must be understood and adapted to dynamic, unexpected, different environments and integrate interdisciplinary staff. A pragmatic approach to data set organization can generate innovative insights in sports sciences and sports teams, guiding practitioners in training, competition, and team member well-being (23).

Some authors describe the constant advancement of sports technology and how AI and Machine Learning (ML) may improve each of these characteristics, injury rates and injury risk (22), athlete health and injuries prevention (25), and maximise sporting performance and athlete well-being (26). Using system thinking, other authors explain how to understand complex systems and their patterns to better understand how they affect sports coaching (27).

Expanding upon a previous investigation conducted in professional and nonprofessional team sports (28), this study describes the present healthcare and performance approaches in elite team sports utilising artificial intelligence. In this update, new domains, applications, or forms of AI use have been identified, as well as new research openings.

### Theoretical framework for AI and ML learning strategies

Artificial Intelligence (AI) and Machine Learning (ML) encompass a broad spectrum of computational techniques designed to emulate intelligent behaviour and facilitate data-based autonomous decision-making. The core objective of ML, a subset of AI, is to enable machines to learn from data, thereby improving their performance on a given task without being explicitly programmed for every scenario.

As will be described below, the most common AI learning strategies in the revised studies include: Tree-based techniques, AdaBoost/XGBoost, Neural Networks, K-Nearest Neighbors (KNN), Classical Regression Techniques, and Support Vector Machines (SVM). In what follows we briefly describe their main characteristics; a more in-depth description can be found in Turing's work (2).

Tree-based techniques, including decision trees and random forests, are useful for their interpretability and ability to handle nonlinear relationships. They are particularly useful for classification and regression tasks.

AdaBoost/XGBoost are boosting algorithms that base their utility on their robustness and efficiency in improving the accuracy of weak learners. They are particularly useful in handling bias-variance trade-offs in predictive models.

The adaptability of neural networks to model complex nonlinear relationships makes them a cornerstone of AI research, particularly in deep learning applications for image and speech recognition, among others.

K-Nearest Neighbors (KNN) shows its simplicity and effectiveness in classification tasks, relying on distance metrics to determine the closest training examples.

Classical Regression techniques remain fundamental for predictive modeling, offering a straightforward approach to understanding relationships between variables.

Finally, Support Vector Machines (SVMs) are suitable in high-dimensional spaces and show their effectiveness in classification problems, especially when data are not linearly separable.

### Objectives

This scoping review aims to provide a global perspective on the current state of the use of artificial intelligence in professional sports teams by:

(a) Identifying the current specific techniques of AI and ML used in sports teams.
(b) Studying the distribution of the selected topics such as sports performance and healthcare in elite sports teams.
(c) Unveiling the sports that are developing these techniques and in which parameters.

## Materials and methods

### Procedures

Our work aligns with those of other authors in healthcare practise and research and uses a scoping approach (29). A scoping review will allow us to summarise research findings from the scientific literature, as well as to identify possible research gaps in a field that needs further research in many aspects. We will use a methodological approach inspired by systematic review methodologies, as suggested by some authors (30). However, there are differences in aims and methods between systematic and scoping reviews, so we will follow the recommendations stated in PRISMA-ScR (31).

A systematic scientific literature search was conducted for this review using the Preferred Reporting Items for Systematic Reviews and Meta-Analyses Extension for Scoping Reviews (PRISMA-ScR) (31). The screening process, extraction process and critical evaluation were done independently by two reviewers: AAMM and MJDM. JLSR supervised all the processes and resolved disagreements to make the last decisions. The same authors carried out the quality evaluation. A web-based repository specifies the initial protocol^1^.

### Querying technique

The Internet databases: Web of Science, Scopus and Pubmed were thoroughly searched from 2019 until the end of 2023 (last 5 years). The various search strings are presented in Table 1. The aim is to analyse the literature of the last five years to ascertain the current state of artificial intelligence applications that attempt to appreciate the intricacies of team sports performance.

**Table 1:** Search strings used in each database and results returned.

Web of science	Scopus	Pubmed
(Elite OR Pro* OR “High Level”) (Topic) and (“Team Sports” OR “Team Sport” OR Football OR Soccer OR Basketball OR Handball OR Volleyball OR Rugby OR “Australian Football” OR Baseball OR “American Football” OR “Ice Hockey”) (Topic) and (“Artificial Intelligence” OR “Computational Intelligence” OR “Machine Intelligence” OR “Computer Reasoning” OR AI OR “Computer Vision System” OR “Machine Learning” OR “Transfer Learning” OR Complexity OR “Complex Model” OR “Big Data” OR “Data Mining” “Agent-Based Modelling” OR “Systems Thinking” OR “System Theory” OR “System Analyses” OR “Artificial Neural Network” OR Bayes OR “Bayesian Logistic” OR “Bayesian Networks” OR “Decision Tree Classifier” OR “Deep Learning” OR “learning algorithms” OR “Neural Network” OR “Random Forest” OR “Support Vector Machine”) (Topic) and (Performance OR Healthcare OR Injury OR “Injury prevention” OR “Injury risk” OR Load OR Control OR “Load control” OR Improvement OR Enhancement OR Management OR Optimize OR Regulation OR Reduction OR Decrease) (Topic) and Article (Document Types) and 2023 or 2022 or 2021 or 2020 or 2019 (Publication Years)	(TITLE-ABS-KEY ((elite OR pro* OR “High Level”)) AND TITLE-ABS-KEY ((“Team Sports” OR “Team Sport” OR football OR soccer OR basketball OR handball OR volleyball OR rugby OR “Australian Football” OR baseball OR “American Football” OR “Ice Hockey”)) AND TITLE-ABS-KEY ((“Artificial Intelligence” OR “Computational Intelligence” OR “Machine Intelligence” OR “Computer Reasoning” OR ai OR “Computer Vision System” OR “Machine Learning” OR “Transfer Learning” OR complexity OR “Complex Model” OR “Big Data” OR “Data Mining” “Agent-Based Modelling” OR “Systems Thinking” OR “System Theory” OR “System Analyses” OR “Artificial Neural Network” OR bayes OR “Bayesian Logistic” OR “Bayesian Networks” OR “Decision Tree Classifier” OR “Deep Learning” OR “learning algorithms” OR “Neural Network” OR “Random Forest” OR “Support Vector Machine”)) AND TITLE-ABS-KEY ((performance OR healthcare OR injury OR “Injury prevention” OR “Injury risk” OR load OR control OR “Load control” OR improvement OR enhancement OR management OR optimize OR regulation OR reduction OR decrease))) AND PUBYEAR > 2018 AND (LIMIT-TO (DOCTYPE, “ar”))	Search: ((((Elite OR Pro* OR “High Level”)) AND ((“Team Sports” OR “Team Sport” OR Football OR Soccer OR Basketball OR Handball OR Volleyball OR Rugby OR “Australian Football” OR Baseball OR “American Football” OR “Ice Hockey”))) AND ((“Artificial Intelligence” OR “Computational Intelligence” OR “Machine Intelligence” OR “Computer Reasoning” OR AI OR “Computer Vision System” OR “Machine Learning” OR “Transfer Learning” OR Complexity OR “Complex Model” OR “Big Data” OR “Data Mining” “Agent-Based Modelling” OR “Systems Thinking” OR “System Theory” OR “System Analyses” OR “Artificial Neural Network” OR Bayes OR “Bayesian Logistic” OR “Bayesian Networks” OR “Decision Tree Classifier” OR “Deep Learning” OR “learning algorithms” OR “Neural Network” OR “Random Forest” OR “Support Vector Machine”))) AND ((Performance OR Healthcare OR Injury OR “Injury prevention” OR “Injury risk” OR Load OR Control OR “Load control” OR Improvement OR Enhancement OR Management OR Optimize OR Regulation OR Reduction OR Decrease)) Filters: in the last 5 years
**1,054**	**225**	**36**

### Criteria for eligibility and the selection process

Two reviewers, AAMM and MJDM, conducted the screening, extraction, and critical assessment processes independently.

We created a thorough selection process to determine eligibility for our study, setting clear inclusion and exclusion criteria with a strong emphasis on the research topic. We started by classifying the titles and abstracts of publications according to their topical significance. When there was uncertainty about a study's significance, author JLSR was asked to make the ultimate decision. Here, we outline these conditions:

The inclusion criteria were created to ensure that the chosen studies were relevant and of good quality. We initially focused on articles written in English to guarantee a widely accessible database. The works must be published as original, comprehensive research articles in peer-reviewed journals between 2019 and the end of 2023 to include up-to-date and pertinent research. We restricted our study to participants in team sports only, omitting those centred on individual sports, to ensure uniformity in our research setting.

The papers must primarily focus on study performance or healthcare in the sports realm to allow us to focus on specific areas of interest. Our interest is on research carried out with elite, professional, or high-level sports teams since these settings provide unique insights into the requirements and results of high performance. It was crucial that the AI methods or algorithms in the studies be well explained and detailed, as this is vital to understand the relevance and efficiency of the suggested solutions.

Exclusion criteria were set to eliminate studies that did not fit our specifications. We excluded research that did not utilise machine learning-based solutions, as our focus was on the implementation of AI in sports. To preserve an emphasis on primary and original research in elite sports teams, studies including mainly non-professional and/or underage players, as well as reviews and meta-analyses, were removed from the research. We excluded study protocols and articles with unavailable full texts to ensure that our review was based on comprehensive and accessible data.

### Quality evaluation

The studies incorporated in the research utilize different observational methodologies: cohort, case-and-control, and cross-sectional. The STROBE checklist (32) was employed to assess the quality of the identified research publications and mitigate any biases (Table 2).

**Table 2:** Item 16 PRISMA ScR protocol: critical appraisal sources of evidence.

First author and year	Title and abstract	Introduction	Methods	Results	Discussion	Conclusion	Funding	Publication bias	Conflict of interest	Adjusted to STROBE	Overall Quality
(33)	Complete	Complete	Complete/CS	Complete	Complete	Complete	Yes	No	No	Yes	High
(34)	Complete	Complete	Complete/CS	Complete	Complete	Complete	Yes	No	No	Yes	High
(35)	Complete	Complete	Complete/CS	Complete	Complete	Complete	No	No	No	Yes	High
(36)	Complete	Complete	Complete/CS	Complete	Complete	Complete	No	No	No	Yes	Moderate
(37)	Complete	Complete	Complete/CS	Complete	Complete	Complete	Yes	No	No	Yes	High
(38)	Complete	Complete	Complete/RCS	Complete	Complete	Complete	No	No	No	Yes	High
(39)	Complete	Complete	Complete/RCS	Complete	Complete	Complete	No	No	No	Yes	High
(40)	Complete	Complete	Complete/CS	Complete	Complete	Complete	No	No	No	Yes	High
(41)	Complete	Complete	Complete/CS	Complete	Complete	Complete	No	No	No	Yes	High
(42)	Complete	Complete	Complete/CS	Complete	Complete	Complete	No	No	No	Yes	High
(43)	Complete	Complete	Complete/CS	Complete	Complete	Complete	No	No	No	Yes	High
(44)	Complete	Complete	Complete/CS	Complete	Complete	Complete	No	No	No	Yes	High
(45)	Complete	Complete	Complete/CC	Complete	Complete	Complete	–	No	–	Partial	High
(46)	Complete	Complete	Complete/CS	Complete	Complete	Complete	Yes	No	No	Yes	High
(47)	Complete	Complete	Complete/CS	Complete	Complete	Complete	Yes	No	Yes	Yes	High
(48)	Complete	Complete	Complete/CS	Complete	Complete	Complete	No	No	No	Yes	High
(49)	Complete	Complete	Complete/CS	Complete	Complete	Complete	Yes	No	No	Yes	High
(50)	Complete	Complete	Complete/CS	Complete	Complete	Complete	No	No	No	Yes	High
(51)	Complete	Complete	Complete/CS	Complete	Complete	Complete	No	No	No	Yes	High
(52)	Complete	Complete	Complete/CS	Complete	Complete	Complete	No	No	No	Yes	High
(53)	Complete	Complete	Complete/RCS	Complete	Complete	Complete	No	No	–	Yes	High
(54)	Complete	Complete	Complete/CS	Complete	Complete	Complete	No	No	No	Yes	High
(55)	Complete	Complete	Complete/CS	Complete	Complete	Complete	–	No	–	Partial	High
(56)	Complete	Complete	Complete/CC	Complete	Complete	Complete	No	No	No	Yes	High
(57)	Complete	Complete	Complete/ERCT	Complete	Complete	Complete	Yes	No	–	Yes	High
(58)	Complete	Complete	Complete/CS	Complete	Complete	Complete	No	No	No	Yes	High
(59)	Complete	Complete	Complete/CS	Complete	Complete	Complete	No	No	No	Yes	High
(60)	Complete	Complete	Complete/CS	Complete	Complete	Complete	Yes	No	No	Yes	High
(61)	Complete	Complete	Complete/CS	Complete	Complete	Complete	No	No	No	Yes	High
(62)	Complete	Complete	Complete/CS	Complete	Complete	Complete	No	No	–	Yes	High
(63)	Complete	Complete	Complete/RCS	Complete	Complete	Complete	No	No	No	Yes	High
(64)	Complete	Complete	Complete/CSS	Complete	Complete	Complete	No	No	No	Yes	High

CS, prospective cohort study; RCS, retrospective cohort study; CC, case-controls; ERCT, experimental randomized controlled trial; CSS, cross-sectional study.

### The main research AI technique or method: classification

#### Topic classification and selection

**Figure 1:**
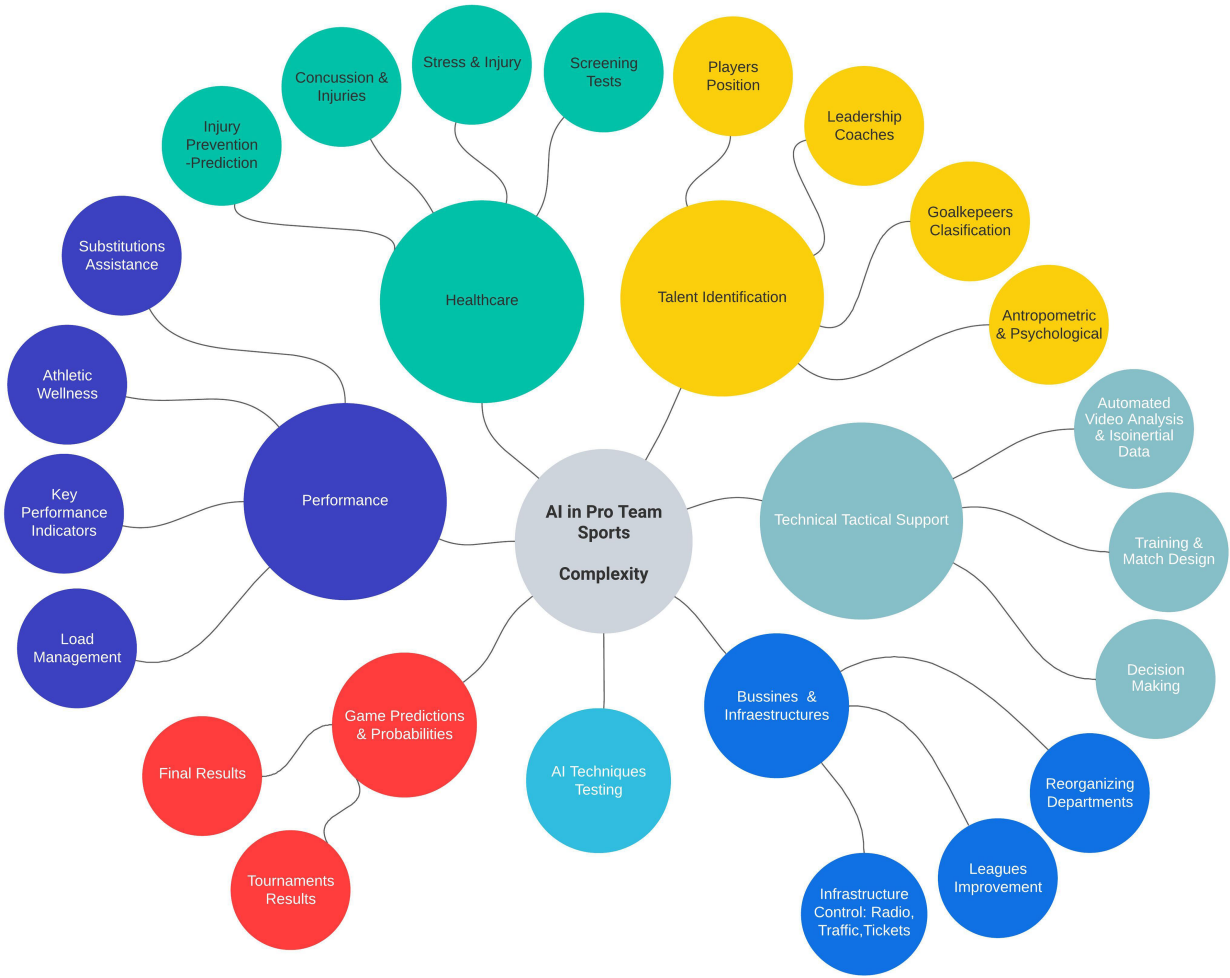
Mental map showing multiple artificial intelligence domains of application in sports teams.

Sports performance, sports healthcare, technical-tactical domain, talent identification, and business domain, among others, were among the domain-specific categories of the application that emerged during the search (Figure 1). The classification and comparison of documents was facilitated by this arrangement. The themes Performance and Healthcare were chosen among others to focus on the aim of the scoping review.

In the Performance section, topics such as the use of GPS for tracking, profiling and decision making, wellness control respecting the load, predicting performance from anthropometric and testing data and key performance indicators (KPI) were explored because of the search and selection results. In the Healthcare section, topics such as injury prevention and prediction with an emphasis on muscle injury processes from injury to return to play, the external load and the injuries, and the integration of psychological, blood biochemical and genetical factors were discussed.

#### AI or ML identified

The key AI learning strategies and methodologies were examined for this extensive analysis and then categorised as follows, depending on their appearance in the studies: Tree-based methods, Ada/XGBoost, Neural Network, K-Nearest Neighbours, Classical Regression Techniques, Support Vector Machine, Naïve-Bayes, Subgroup Discovery and Clustering Analysis were ordered by frequency of appearance. Then, the investigations were categorised into this fundamental learning strategy or method and the comprehensive method as part of the investigation into the complexities and procedures of artificial intelligence.

The metrics used to evaluate the model depended on the AI technique used. As a rule, to order the models, we designated the one that exhibited the most optimal performance when various techniques were used in the same study.

#### Metrics for evaluation

The evaluation of these AI learning strategies employs several metrics to assess their performance comprehensively:

*True Positive (TP), False Positive (FP), True Negative (TN), False Negative (FN)*.

*Accuracy:* Measures the proportion of correct predictions among the total number of cases examined. Proportion of TP and TN in all evaluated cases (see Equation 1).

*Sensitivity (or Recall):* Assesses the ratio of true positive predictions to the actual positives. Proportion of TP in all the cases that belong to this class (see Equation 2).

*Specificity:* proportion of TN in all cases that don't belong to this class (see Equation 3).

*Precision:* Evaluates the ratio of true positive predictions to the total positive predictions. Proportion of TP in all cases that have been classified as it (see Equation 4).

*F1-score:* Provides a harmonic mean of precision and recall, balancing the two metrics. A measure of a test's accuracy. It considers both the precision and the sensitivity (recall) of the test to compute the score. It is the harmonic mean of both parameters (see Equation 5).

*Area Under the Receiver Operating Characteristic Curve (AUC-ROC):* This metric gauges the model's ability to distinguish between classes.

*The root mean square (RMS)* measure in artificial intelligence studies refers to the square root of the average of the squares of a set of values. In the AI context, it is used to calculate the square root of the average of the squared errors between the values predicted by a model and the actual values of the data.

*Root Mean Square Error (RMSE):* Similarly to RMS, RMSE is the square root of the average of the squared differences between predicted values and actual values. It gives a measure of the magnitude of the error.

*Logarithmic Loss (Log Loss):* This metric measures the performance of a classification model where the prediction is a probability value between 0 and 1. Log loss increases as the predicted probability diverges from the actual label.


(1) $$accuracy=\underset{c}{\sum }\frac{TP_{c}+\;TN_{c}}{TP_{c}+\;TN_{c}+\;FP_{c}+\;FN_{c}}\;,c\;\epsilon \;classes$$

(2) $$sensitivity=\underset{c}{\sum }\frac{TP_{c}}{TP_{c}+\;FN_{c}},\;c\;\epsilon \;classes$$

(3) $$specificity=\underset{c}{\sum }\frac{TN_{c}}{TN_{c}+\;FP_{c}},\;c\;\epsilon \;classes$$

(4) $$precision=\underset{c}{\sum }\frac{TP_{c}}{TP_{c}+\;FP_{c}},\;\;c\;\epsilon \;classes$$

(5) $$F1_{score}=2*\frac{precision*sensitivity}{precision+sensitivity}$$

## Results

**Figure 2:**
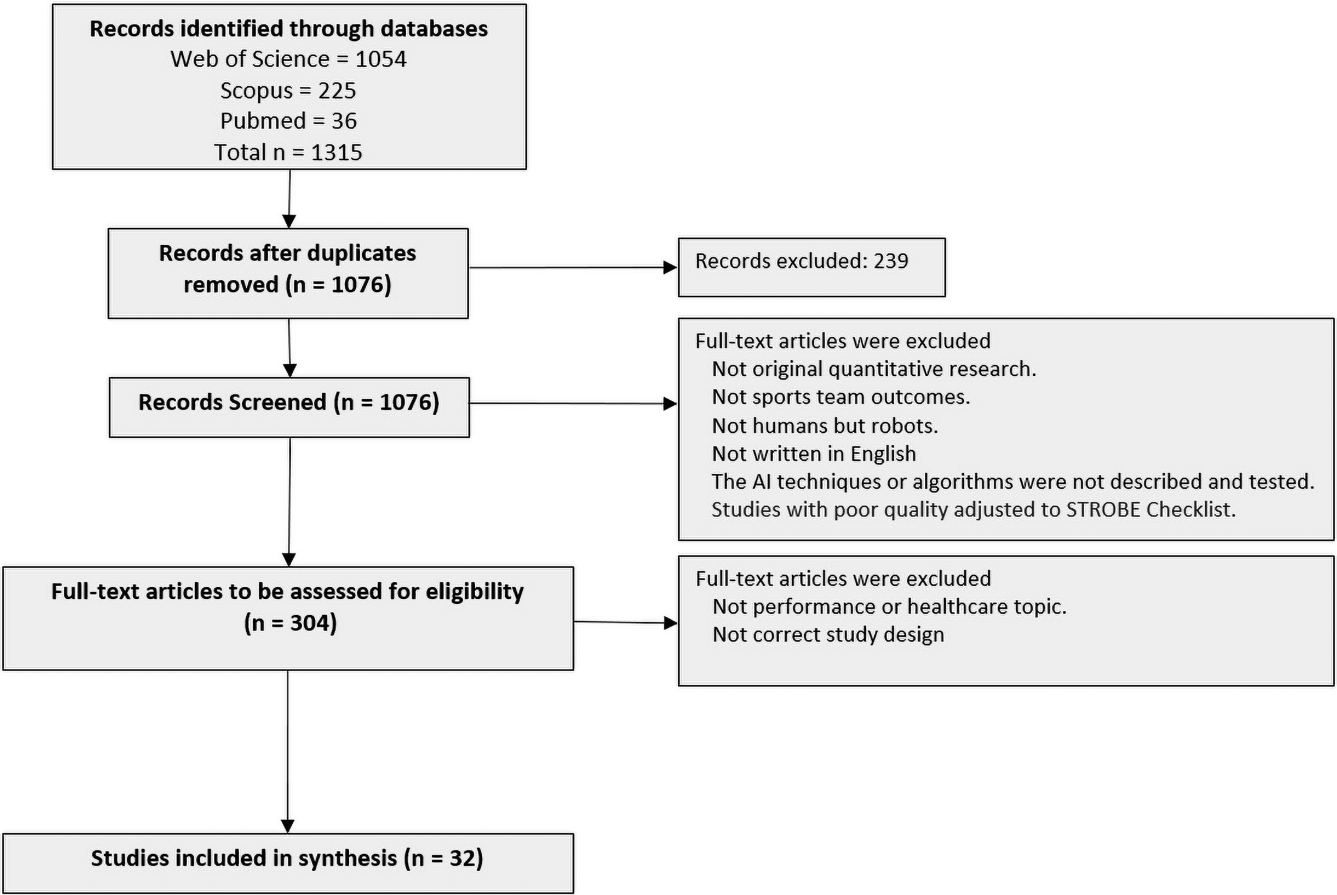
PRISMA-ScR protocol: selection of sources of evidence, item 14.

The search found 1,315 articles as the initial selection of sources of evidence (Figure 2). After deleting duplicate articles, we obtained 1,076 research papers. After applying the inclusion and exclusion criteria we obtained 32 studies that were considered for full-text evaluation.

During the selection process, a rather curious discovery was made: we found that some studies (*n* = 21) corresponded to elite team competitions involving robots and not people. They were not included in our review.

Afterwards, some articles remained, and a deep reading revealed that the AI technique or the algorithms were not described, and applying the risk of bias threshold to the studies some of them were discarded.

The results summarizing the population and the AI or ML used in the selected studies can be found in Table 3.

**Table 3:** Item 17 PRISMA ScR protocol: results of individual sources of evidence.

Author	Population	IA or machine learning technique approach identified
(33)	18 elite soccer players—age = 24.7 (4.3) years; height = 183.73 (7.16) cm; weight = 78.81 (7.32) kg—competing in the Italian league (Serie B) during the seasons 2017/2018 and 2018/2019 took part in this study—128 (36) sessions per player.	Players’ blood samples’ characteristics permitted to personalize of the decision-making rules of the ML models based on external workloads reaching an accuracy of 63%. This approach increased the injury prediction ability by about 15% compared to models that take into consideration only training workloads’ features. Clustering and Multidimensional Model were used
(34)	112 professional male adult football (soccer) players from 4 teams of the German 4th and 3rd leagues have participated of whom 88 could be included in the analysis.	The cross-validated performance of the gradient boosted model (ROC area under the curve 0.61) was promising and higher compared to models without integration of screening data. Importantly, holdout test set performance was similar (ROC area under the curve 0.62) indicating the prospect of generalizability to new cases.
(35)	36 Male professional players average age of 24 (5.26 SD). The club KKS Lech	Three decision-making methods were implemented. Rule-based and fuzzy rule-based methods were prepared based on expert understanding. Machine learning baseline was done with XGBoost algorithm.
(36)	21 Elite female soccer team prior to the competitive season.	Dimension reduction through linear principal component analysis (PCA), non-linear kernel principal component analysis (k-PCA), t-distributed stochastic neighbour embedding (t-sne), and uniform manifold approximation and projection (umap) for injury markers via grid search
(37)	38 elite soccer players aged 19-27 years were observed during 151 training sessions, 44 matches across a full season.	Machine learning models (linear regression, K-NN, decision trees, random forest, elastic net regression, XGBoost) were compared and interpreted to deepen the relationship between external load variables and ratings of perceived exertion according to the player position in a predictive perspective.
(38)	77 professional male football players were assessed at the start of the season (baseline) and, respectively, at 4, 3, 2 and 1 weeks before the injury.	A data mining technique named Subgroup Discovery, is a descriptive, exploratory technique that can handle relatively small datasets.
(39)	64 elite soccer players were monitored during an entire season	Extreme gradient boosting (XGBoost), Random Forest Regression (RF), Linear Regression (LR)
(40)	41 unique male soccer players (mean age 24 ± 4.224 ± 4.2 years)	Integrating the expert's predictions as a feature improves the performance of all models, with XGB performing best with a mean *R*^2^ score of 0.720, outperforming the expert's predictions with an *R*^2^ score of 0.62
(41)	14 elite volleyball players (mean ± SD age: 27 ± 3 years, weight: 90.5 ± 6.3 kg, height: 1.97 ± 0.07 m)	The machine learning technique Subgroup Discovery.
(42)	All male professional football players from FC Barcelona, with injuries that occurred between February 2010 and February 2020 were reviewed	Three different statistical and machine learning approaches (linear regression, random forest, and eXtreme Gradient Boosting) were used to assess the importance of each factor of the MLG-R classification system in determining the return to play, as well as to offer a prediction of the expected return to play
(43)	36 male elite football players who competed in the First Portuguese Soccer League in the 2020/2021 season	This study considered multiple-input single-output regression-type models. The analysis showed that the most accurate model presented in this work generates an error of RMSE = 0.591.
(44)	25 elite male volleyball players volunteered to participate in this study (mean ± SD age: 27.0 ± 3.0 years, weight: 91.2 ± 6.4 kg, height: 2.00 ± 0.10 m). All subjects competed on the international level	Machine learning techniques XGBoost, random forest regression and subgroup discovery
(45)	791 female elite handball and soccer players	For the best classifier (linear support vector machine), the mean AUC-ROC was 0.63. AUC-ROC values varied substantially across repetitions and methods (0.51–0.69). Class imbalance handling did not improve the results.
(46)	42 professional football players. FC Lucerne football club occurred during 1.5 seasons (2019–2021).	Definition of models and multivariate approaches: Time-series forecasting, Ridge regularisation, Long short term memory neural network
(47)	17 players competing in the Italian championship (Serie A)	Cross-validation and real scenario approaches. The cross-validation approach randomly splits the dataset in train and test sets, while the real scenario approach continuously creates train and test as the season goes.
(48)	38 male soccer players (age 25 ± 3 years; height 1.81 ± 0.06 m; weight 76 ± 5 kg) that played at least one game during the acquisition period	Clustering Analysis
(49)	30 soccer players (Forwards = 7, Midfielders = 14, Defenders = 9, Age = 25.97 ± 4.39, Height = 181.07 cm ± 5.54 cm, Weight = 73.20 ± 6.39 kg) of a team in K League 1, during the 2019 season	Various models were used to generate baseline: Random Forest, Lightgbm, gradient Boosting Regressor, K-Nearest Neibor, Decision Tree, Adaboost Regressor, Linear Regressor, Ridge Regressor, Bayesian Ridge, Elastic Net, Lasso Regressor. The proposed model, named FatigueNet, effectively predicted the RPE with mean absolute error (MAE) = 0.8494 ± 0.0557 and root mean square error (RMSE) = 1.2166 ± 0.0737 using the preprocessed movement features.
(50)	46 European Super League Rugby Players (ESL) 36 under-19 Academy (Academy) level matches over two seasons. 31 ESL players and 41 Academy players participated	Resulting in 157 predictor variables. Data were split into training and testing datasets. Random forests (RF) were built to reduce the dimensionality of the data, identify variables of importance and build classification models. To aid practical interpretation, conditional inference (CI) trees were built. Nine variables were identified as most important for backs, classifying between levels with 83% (RF) and 78% (CI tree) accuracy.
(51)	28 subelite players [age = 20.9 (2.4) years; height = 181.0 (5.8) cm; body mass = 72.0 (4.4) kg] throughout the 2017/2018 season.	A higher TL was reported after the games and during match day (MD)-5 and MD-4, while a higher WI was recorded on the following days (MD-6, MD-4, and MD-3, respectively). A significant correlation was reported between daily TL (TLMDi) and WI measured the day after (WIMDi + 1) (*r* = .72, *P* < .001). Additionally, a similar weekly pattern seems to be repeating itself throughout the season in both TL and WI. Nevertheless, the higher accuracy of ordinal regression (39% [2%) compared with the results obtained by baseline B1 (21% [1%) demonstrated that the machine learning approach used in this study can predict the WI according to the TL performed the day before (MD < i).
(52)	139 [72 (age: 22.5 ± 5.2 years, stature: 1.75 ± 0.7 m, body mass: 72.9 ± 6.9 kg) males and 67 (age: 22.4 ± 5.5 years, stature: 1.64 ± 0.5 m, body mass: 59.4 ± 5.1 kg) females] elite futsal players from 12 different teams [56 players (24 males and 32 females) from six club engaged in the First (top) National Spanish Futsal division and 83 players (48 males and 35 females) from six clubs engaged in the Second National Futsal division] completed this study	Four classifiers based on different paradigms, namely decision trees with C4.5 and ADTree, Support Vector Machines with SMO and the well-known k-Nearest Neighbor (KNN) as an Instance-Based Learning approach were selected. The configuration of each base classifier was optimized through the use of the metaclassifier MultiSearch.
(53)	Retrospectively collected data from the in-match position tracking data from 302 competitive professional soccer matches between 18 teams during the Dutch “Eredivisie” 2018–2019 season.	Machine learning models used individualised normalised variables to predict whether players will perform at 100%, 95%, or 90% of their average physical performance in a match. Models were built using Random Forest and Decision Tree. Simple Naïve Bayes was used as a baseline model to demonstrate the superiority of tree-based algorithms. Machine learning method Random Forest and power category variable energy expenditure were most accurate. The combination of Random Forest and power category energy expenditure predicted performance and underperformance after 15 min in a match with precision of 0.91, 0.88, and 0.92 for 100%, 95%, and 90% thresholds, respectively. To conclude, early match predictions can be made about player physical performance. These findings can help elite soccer coaches make better substitution decisions.
(54)	40 players (mean ± SD; age 29.4 ± 5.8 years; height 175.3 cm ± 5.2 cm; body mass 76.5 ± 8.2 kg) from the same elite soccer club competing in the French Ligue 2 participated in one full-season (2017/2018) data collection	Various classification machine-learning algorithms that performed best on external and internal loads features were compared using standard performance metrics such as accuracy, precision, recall and the area under the receiver operator characteristic curve. In particular, tree-based algorithms based on non-linear models with an important interpretation aspect were privileged as they can help to understand internal and external load features impact on injury risk
(55)	59 female players (age: 20.7 ± 5.4 years, height: 164.0 ± 6.7 cm, bodyweight: 62.8 ± 10.0 kg and BMI: 23.2 ± 2.7 kg.m^2^) from the North Cyprus Women's Handball Super League.	The results showed that the radial-basis function neural network outperformed the other models and was capable of predicting the studied types of athletic performance with *R*^2^ scores between 0.86 and 0.97. Finally, significant factors influencing predicted performance were determined by retraining the superior model. This is one of the first studies using machine learning in sport sciences for handball players, and the results are encouraging for future studies.
(56)	363 [mean (SD); 25 (6) years, 89% male] elite players (soccer, futsal, basketball, handball, and roller hockey) from a top-level European team (FC Barcelona, Spain). Of 363, 55% (cases) had experienced 1+ episodes of tendinopathy during 2008–2018 and 45% (controls) remained injury free.	Machine learning-based multivariate modelling (support vector machine and random forest) to build a reliable predictive model.
(57)	14 professional male soccer players, from a Polish professional soccer club, they had a mean ± SD age of 23.2 ± 2.7 years (age range: 19–27 years), height of 178 cm ± 6 cm, body mass of 73.2 ± 6.9 kg, body fat of 12.6% ± 2%, and 14 ± 5 years of soccer experience	Decision tree induction was applied to the dataset to assess the cut-off point-values from four training drills (SSG, LSG, MG, and CT) and FM for every parameter combination.
(58)	26 professional players were assessed during a competitive Australian Football League (AFL) season	Parametric and machine-learning analysis techniques found several indices of physical load associated with muscle damage during competition, with impacts >3 g and high-intensity running variables as the strongest predictors. Generalized estimating equations and random forest models were constructed
(59)	96 male professional soccer players took part in the current study. Soccer players were recruited from 4 different soccer teams that were engaged in the 1st (one team, *n* = 25) and 2nd B (3 teams, *n* = 73) Spanish National Soccer League divisions.	There were 18 HSIs. Injury distribution was 55.6% dominant leg and 44.4% nondominant leg. The model generated by the SmooteBoostM1 technique with a cost-sensitive ADTree as the base classifier reported the best evaluation criteria (area under the receiver operating characteristic curve score = 0.837, true positive rate = 77.8%, true negative rate = 83.8%) and hence was considered the best for predicting HSI. The model generated by the SmooteBoostM1 technique with a cost-sensitive ADTree as the base classifier reported the best evaluation criteria (area under the receiver operating characteristic curve score
(60)	26 professional male soccer players (mean ± SD age: 23.2 ± 3.7 years, weight: 77.5 ± 7.4 kg, height: 1.82 ± 0.06 m, body fat: 10.4% ± 1.9%) competing for the same team at the highest level in the Netherlands were collected during the 2015–2016 season, both pre-season and in-season	Predictive models were constructed using gradient-boosted regression trees (GBRT) and one naive baseline method
(61)	22 elite soccer players (age = 21.96 ± 4.53 years; height = 180.68 cm ± 5.23 cm; weight = 72.36 ± 4.19 kg) competing in an Italian championship during the 2016/2017 season were recruited in this study	Machine learning applied to training workloads performed in the previous week have a strong effect on perceived exertion and training load. On the other hand, the analysis of our predictions shows higher accuracy for medium RPE and S-RPE values compared with the extremes
(62)	37 Australian football players (age: 23 ± 4 years, height: 187 cm ± 8 cm, mass: 86 ± 9 kg).	Segment similarity for each quotient was evaluated using a random forest model. The strongest classification features in the model were spectral entropy and skewness. Offensive and defensive involvements were the weakest features for classification, suggesting skilled output is dependent on match circumstances
(63)	56 matches across the 2017 and 2018 Australian Football League Women's (AFLW) seasons	Generalised estimating equations (GEE) and regression decision trees were run across the different feature sets and dependent variables, resulting in 22 separate models.
(64)	44 elite male futsal players	A feature selection process was carried out before building a BN (using the Tabu search algorithm) for each leg

Finally, Table 4 gives the results of individual sources of evidence, and shows the synthesis of results, summarizing and presenting the charting results as they relate to the review questions and objectives of the research.

**Table 4:** Item 18 PRISMA ScR protocol: synthesis of results.

Reference, first author, date	Country national or regional	Date of collection, survey, recruitment	Analysis	Analyzed, *n*.	Data Collection Method or Behavior Analyzed	Sport	Results	Category of this article
(33)	Italia	Seasons 2017/2018 and 2018/2019. Italian League (Serie B)	Prospective observational cohort study	18 male elite soccer players—age = 24.7 (4.3) years; height = 183.73 (7.16) cm; weight = 78.81 (7.32) kg—	128 (36) sessions per player. Blood parameters, internal workload: Hematocrit, Hemoglobin, number of red blood cells, ferritin, and sideremia External workload data recorded every training or match day using a GPS device. (K-GPS 10 Hz, K-Sport International, Italy)	Football	Players’ blood samples’ characteristics permitted to personalize the decision-making rules of the ML models based on external workloads reaching an accuracy of 63%. This approach increased the injury prediction ability of about 15% compared to models that take into consideration only training workloads’ features. Clustering and Multidimensional Model were used.	P/H (Performance and Health) Healthcare
(34)	Germany	The 2019/2020 football season	Prospective observational cohort study	88 male players from 4 teams and 51 injuries could be analysed	Gradient boosting with ROSE upsampling within a leave-one-out cross-validation was used for data analysis.	Football	Gradient boosted model with cross-validated performance of ROC area under the curve 0.61	P/H (Performance and Health) Healthcare
(35)	Poland	Autumn round of the 2020/2021 season	Prospective observational cohort study	36 male players with an average age of 24 (±5.26 SD)	Predicting non-contact lower body injuries coming from over or undertraining by GPS Data	Football	Machine learning method XGBoost algorithm (Precision 92.4%, Recall 96.5%, and F1-score 94.4%).	P/H (Performance and Health) Healthcare
(36)	Chile	Season 2021/2022	Prospective observational cohort study	21 female elite soccer athletes (age: 22.5 ± 5.5 years, body mass: 61.3 ± 8.3 kg, height: 1.62 ± 0.06 m, and body mass index: 23.3 ± 2.9 kg m^−2^	To determine the influence of dimension reduction for pattern recognition followed by clustering on multiple biomechanical injury markers in elite female soccer players during preseason. Muscle strength, muscle function, jump technique and power, balance, muscle stiffness, exercise tolerance, and running performance were assessed in an elite female soccer team	Football	Umap facilitated the injury pattern recognition compared to PCA, k-PCA, and t-sne. One of the three patterns was related to a team subgroup with acceptable muscle conditions. In contrast, the other two patterns showed higher injury risk profiles. For our dataset, umap improved injury surveillance through multiple testing characteristics.	P/H (Performance and Health) Healthcare
(37)	France	151 training sessions and 44 matches across a full season	Prospective observational cohort study	38 male players (mean ± SD age: 23.4 ± 4.2 years, height: 180.7 cm ± 6 cm, body mass: 77.4 ± 6.8 kg) participating in the French Ligue2 championship	External load variables (58 derived from Global Positioning System and 30 from accelerometers) and the internal load derived from ratings of perceived exertion were collected for each player and each session and match.	Football	The ML models predicted HCE severity with an average area under the receiver operating characteristic curve of 0.73 for male and 0.70 for mixed datasets. The injury mechanism and pre-injury incident were most predictive. The mechanisms “head-to-head” and “knee-to-head” were significantly linked with severity in the male dataset (*P* = 0.0244) but not in the mixed sample (*P* = 0.1113). Both datasets showed that “corner kicks” and “throw-ins” were linked with severity (male, *P* = 0.0001; mixed, *P* = 0.0004).	P/H (Performance and Health) Healthcare
(38)	Belgium	2020–2021 season to November 2022	Retrospective observational cohort study	77 male professional players from the highest Belgian league.	77 professional male football players were assessed at the start of the season (baseline) and, respectively, at 4, 3, 2 and 1 weeks before the injury. We included 278 cases (92 injuries; 186 healthy) and applied a subgroup discovery algorithm. Studied: Eccentric hamstring strength, Isometric hip adductor and abductor strength Countermovement jump	Football	More injuries occurred when between-limb abduction imbalance 3 weeks prior (threshold ≥0.97) or right leg adduction muscular strength 1 week prior (threshold ≤1.01) remained unchanged or deteriorated. In 50% of instances, an injury occurred if abduction strength imbalance was over 97% of baseline values and peak landing force in the left leg was less than 124%.	P/H (Performance and Health) Healthcare
(39)	Italia	The 2022/2023 soccer season.	Prospective observational cohort study	64 elite male players from the first team (n: 24, age: 23.9 ± 4.7, body mass: 80.0 ± 6.5, height: 184.6 ± 5.4), U19 team (*n*: 19, age: 18.4 ± 0.7, body mass: 75.5 ± 8.8, height: 181.3 ± 6.8), and U1	Players’ external load was recorded during 511 training sessions (first team, *n* = 199, duration: 67.7 ± 14.1 min; U19: 166, duration: 85.6 ± 18.2 min; U18: 146, duration: 85.6 ± 13.5 min) and 116 official matches (first team, *n* = 38; U19, *n* = 40; U18, *n* = 38). An average of 169 ± 31 observations per player were recorded.	Football	The Random Forest Regression (RF) performed best (mean absolute percentage error = 0.10 ± 0.01) and was used in further investigations. Using a z-score transformation (LEI), the difference between the ML model's anticipated PL value and the real one was determined, customised for each player, and interpreted as weariness (negative LEI) or neuromuscular preparedness (positive LEI).	P/H (Performance and Health) Healthcare
(40)	Croatia	February 2021 to February 2023	Prospective observational cohort study	41 unique male soccer players (mean age 24 ± 4.2 years)	The focus of this study was to estimate muscle injuries, which were classified using the British Athletic Muscle Injury Classification (BAMIC). A total of 84 muscle injuries were studied during the examined period.	Football	The results demonstrate that integrating the expert's predictions as a feature improves the performance of all models, with XGB performing best with a mean *R*^2^ score of 0.720.72, outperforming the expert's predictions with an *R*^2^ score of 0.62	P/H (Performance and Health) Healthcare
(41)	Netherlands	During 24 weeks of the 2018 international season	Prospective observational cohort study	14 elite male volleyball players (mean ± SD age: 27 ± 3 years, weight: 90.5 ± 6.3 kg, height: 1.97 ± 0.07 m).	Training sessions and matches were monitored during 24 weeks of the 2018 international volleyball season for national teams. The number of days on which a player is monitored was 94 ± 18 Monitored: Injuries, illness and severity of complaints, Perceived wellness, Training load.	Volleyball	Professional volleyball players completed a total of 1,112 questionnaires, and in 313 cases (28.1%), the players reported overuse complaints, i.e., severity score >0 for at least one of the four questions. In 25 entries (2.2%), athletes reported substantial complaints (severity score ≥50) for affected performance (Q3) or reduced training volume (Q2)	P/H (Performance and Health) Healthcare
(42)	Spain	From 2010 to 2020	Retrospective observational cohort study	76 hamstring injuries corresponding to 42 different players.	76 hamstring injuries corresponding to 42 different players were identified, of which 50 (65.8%) were grade 3rd, 54 (71.1%) affected the biceps femoris long head, and 33 of the 76 (43.4%) were located at the proximal myotendinous junction	Football	The statistical analysis showed an excellent predictive power of the MLG-R classification system with a mean absolute error of 9.8 days and an R-squared of 0.48. The most important factors to determine the return to play were if the injury was at the free tendon of the biceps femoris long head or if it was a grade 3^r^ injury. For all the items of the MLG-R classification, the intra-observer and inter-observer reliability was excellent (*k* > 0.93) except for fibres blurring (*κ* = 0.68).	P/H (Performance and Health) Healthcare
(43)	Portugal	First Portuguese Soccer League in the 2020/2021 season	Prospective observational cohort study	36 male elite football players	22 independent variables—player information, body composition, physical fitness, and season injuries—were used to calculate the models. In the net elastic analysis, defensive and forward sectorial positions, body height, sit-and-reach performance, 1 min push-ups, handgrip strength, and 35 m linear speed best predicted injury risk.	Football	Classic regression models (OLS), shrinkage regression, and stepwise regression were used in the models’ calculations. The analysis showed that the most accurate model presented in this work generates an error of RMSE = 0.591.	P/H (Performance and Health) Healthcare
(44)	Netherlands	National team stages during the 2018 international volleyball season	Prospective observational cohort study	25 elite male volleyball players volunteered to participate in this study (mean ± SD age: 27.0 ± 3.0 years, weight: 91.2 ± 6.4 kg, height: 2.00 ± 0.10 m).	Over 24 weeks, all training sessions and matches, as well as daily wellness, were monitored. The players participated in 6.1 ± 2.4 training sessions per week and trained for 13.8 ± 5.0 h per week. In total, the players competed in 31 matches, including 17 friendly matches.	Volleyball	Random Forest, XGBoost and Action Model were used to determine the performance in the defense and offense game phases	P/H (Performance and Health) Performance
(45)	Norway	Between the years 2007 and 2015	Case-control study;	791 female elite 451 soccer and 429 handball players (age, 21 ± 4 years; height, 170 cm ± 6 cm, weight, 66 ± 8 kg)	Investigate the predictive potential of multiple predictive machine learning methods on a large set of risk factor data for anterior cruciate ligament (ACL) injury; the proposed approach takes into account the effect of chance and random variations in prediction performance.	Handball and Football	For the best classifier (linear support vector machine), the mean AUC-ROC was 0.63. AUC-ROC values varied substantially across repetitions and methods (0.51–0.69).	P/H (Performance and Health) Healthcare
(46)	Switzerland	FC Lucerne football club over a 1.5 season period (2019–2021)	Prospective observational cohort study	42 male players.	This study aims to predict individual Acceleration-Velocity profiles (A-V) from Global Navigation Satellite System (GNSS) measurements in real-world situations.	Football	Multivariate models were fitted per player or per group. GNSS characteristics were weak predictors of individual A-V profiles.	P/H (Performance and Health) Performance
(47)	Italia	Season 2016/2017	Prospective observational cohort study	17 male players (age = 23.35 ± 5.63 years; height = 182.17 cm ± 6.40 cm; weight = 80.91 ± 8.34 kg)	Italia championship (Serie A). The club tracked players’ GPS (Viper Units 10 Hz, STATSports, Newry, Ireland) during training and matches. The season had 2,728 sessions, averaging 160.47 ± 34.54 per player.	Football	At the end of the season, XGB shows the higher cumulative performance goodness (accuracy = 0.63) compared to DTC (accuracy = 0.56) and B_s_ (accuracy = 0.37). We find that XGB's accuracy increases as the weeks go by. Actually, in the last week, XGB reached 87% accuracy.	P/H (Performance and Health) Performance
(48)	Croatia	Two half-seasons in 2021 and 2022 and included 80 games	Prospective observational cohort study	38 male soccer players (age 25 ± 3 years; height 1.81 ± 0.06 m; weight 76 ± 5 kg) that played at least one game during the acquisition period.	Before each game, players would put on the GPexe pro2 device,. After the match, collected data were downloaded. This included: total time played (min), distance (m), average metabolic power (W/kg), energy (J/kg), anaerobic energy (J/kg), MPE count, MPE average recovery time (s), MPE average recovery power (W/kg), walk distance (m), running distance (m), walk energy (J/kg), and running energy (J/kg).	Football	K-means was selected to evaluate how many intensity zones were present in the dataset. Our framework exhibits a higher explanatory power compared to usual game metrics (e.g., high-speed running and sprinting), explaining 45.91% of the coefficient of variation vs. 21.32% for high-, 30.66% vs. 16.82% for middle-, and 24.41% vs. 19.12% for low-intensity periods.	P/H (Performance and Health) Performance
(49)	Korea	2019/2020 season	Prospective observational cohort study	30 male soccer players (Age = 25.97 ± 4.39, Height = 181.07 cm ± 5.54 cm, Weight = 73.20 ± 6.39 kg) of a team in K League 1, which is the first division of the Korean professional soccer league	K League 1 Korean professional soccer league provided data for the investigation. The gadgets recorded locations from 163 training sessions and 39 matches. Players must record RPE without anchoring after each activity. They rated the activity's difficulty from 1 to 10.	Football	Various models were used to generate baseline: Random Forest, Lightgbm, gradient Boosting Regressor, K-Nearest Neibor, Decision Tree, Adaboost Regressor, Linear Regressor, Ridge Regressor, Bayesian Ridge, Elastic Net, Lasso Regressor. The proposed model, named FatigueNet, effectively predicted the RPE with mean absolute error (MAE) = 0.8494 ± 0.0557 and root mean square error (RMSE) = 1.2166 ± 0.0737 using the preprocessed movement features.	P/H (Performance and Health) Performance
(50)	United Kingdom	Not specified	Prospective observational cohort study	31 male ESL players (height: 186.0 cm ± 7.0 cm, body mass: 98.28 ± 10.73 kg, age: 27.3 ± 4.8 years) and 41 male Academy players (height: 178.6 cm ± 6.4 cm, body mass: 90.1 ± 13.2 kg, age 17.7 ± 1.0 years)	Microtechnology units were used to analyse the physical PI and matches were videoed and coded for individual technical-tactical PI, resulting in 157 predictor variables. Match observations per player were 22 ± 13 (range: 1–43) and 14 ± 9 (range: 1–36)	Rugby	The factors with the highest classification rate were PlayerLoad2D, PlayerLoadSLOW per Kg body mass, and high-speed running distance. Four characteristics were most relevant for forwards, categorising with 68% (RF) and 64% (CI tree) accuracy. Forwards’ highest classification rate was defensive play-the-ball losses.	P/H (Performance and Health) Performance
(51)	Italia	2017/2018 season	Prospective observational cohort study	28 subelite male players [age = 20.9 (2.4) y; height = 181.0 (5.8) cm; body mass = 72.0 (4.4) kg] throughout	Predictive models were constructed using a supervised machine learning method that predicts the WI according to the planned TL. The validity of the predictive model was assessed by comparing the classification's accuracy with the one computed from a baseline that randomly assigns a class to an example by respecting the distribution of classes (B1).	Football	TL and WI were higher after the games and on match days (MD)-5 and MD-4, respectively (MD-6, MD-4, and MD-3, respectively). A strong association exists between daily TL (TLMDi) and the day-after WI (WIMDi + 1) (*r* = .72, *P* < .001). In both TL and WI, a similar weekly trend appears throughout the season. Higher accuracy of ordinal regression [39 percent (2 percent)] compared to baseline B1 [21 percent (1 percent)] suggests the machine learning approach utilised in this study can predict WI based on the previous day's TL (MD < i).	P/H (Performance and Health) Performance
(52)	Spain	End of the pre-season phase in 2015 (39 players from four teams), 2016 (44 players from four teams), 2017 (30 players from three teams), and 2018 (26 players from two teams) (September)	Prospective observational cohort study	139 elite futsal players from 12 teams consisted of 72 males and 67 females. The sample included 56 players (24 males and 32 females) from six clubs in the First National Spanish Futsal division and 83 players (48 males and 35 females) from six clubs in the Second	Personal or Individual Measures Psychological Risk Factors Self-Perceived Chronic Ankle Instability Neuromuscular Risk Factors Injury Surveillance	Football Indoor	Four field-based screening models (mainly ROM and dynamic postural control features) with moderate accuracy (AUC scores ranged from 0.701 to 0.767, determined using the rigorous cross-validation resampling technique) for identifying elite futsal players at risk of LE-ST injury were developed in this study. Only two hip (flexion with knee extended and abducted) and two ankle (dorsiflexion with knee flexed and extended) ROM measures and 10 classifiers made up the current study's “model of best fit” (AUC = 0.767, TP rate = 85% and TN rate = 62%).	P/H (Performance and Health) Healthcare
(53)	Netherlands	2018–2019 season	Retrospective observational cohort study	480 male players participated in the 300 matches	Power and Performance variables are classified as type 1 and type 2. Four thousand nine hundred and thirty-five times, entire-match players were identified. In addition, 1,533 substitutes were identified.	Football	Our study showed that position monitoring system type 1 and type 2 variables could identify physical performance. Both type 1 and type 2 variables favour substitutions over entire-match players. Machine learning predicts a player's physical performance early in the match using the more sensitive type 2 variable, which is more precise than type 1.	P/H (Performance and Health) Performance
(54)	France	French Ligue 2 participated in one full-season (2017/2018)	Prospective observational cohort study	40 male players (mean ± SD; age 29.4 ± 5.8 years; height 175.3 cm ± 5.2 cm; body mass 76.5 ± 8.2 kg)	Training workload, perceptive well-being questionnaires, and injury data were observed from June 2017 to May 2018, taking into account international truces and the winter ceasefire. The analysis included 245 training sessions, 38 Domino's Ligue 2 matches, 2 Coupe de la Ligue matches, and 3 Coupe de France matches.	Football	Comparing classification machine-learning algorithms that performed best on external and internal loading features by accuracy, precision, recall, and area under the receiver operator characteristic curve. Tree-based algorithms based on non-linear models with an essential interpretation aspect were favoured because they can help comprehend how internal and external load variables affect injury risk.	P/H (Performance and Health) Healthcare
(55)	Chipre	Not defined	Prospective observational cohort study	59 female players (age: 20.7 ± 5.4 years, height: 164.0 cm ± 6.7 cm, bodyweight: 62.8 ± 10.0 kg and BMI: 23.2 ± 2.7 kg.m^2^) from the North Cyprus Women's Handball Super League	In total, 23 properties were comprises comprising Anthropometric measures, power tests (Wingate) and jump tests (CMJs)	Handball	The study found that the radial-basis function neural network accurately predicted athletic performance with *R*^2^ scores ranging from 0.86 to 0.97, outperforming previous models. Finally, retraining the improved model identified performance-predicting factors.	P/H (Performance and Health) Performance
(56)	Spain	Seasons from 2008 to 2018	Case-Controls	363 elite soccer, futsal, basketball, handball, and roller hockey players [mean (SD); 25 (6) years, 89 per cent male] (FC Barcelona, Spain). Of 363, 55% (cases) had 1+ tendinopathy episodes in 2008–2018, while 45% (controls) were injury-free.	The authors first assessed the connection between single-nucleotide polymorphisms (SNPs) and tendinopathy risk in a hypothesis-free case-control genome-wide association research (495,837 SNPs) with target analysis of 58 SNPs identified as potential risk factors in the literature. After synthetic variant imputation (1,419,369 SNPs), the authors built a reliable predictive model using machine learning-based multivariate modelling (support vector machine and random forest).	Multisport	Some suggestive associations were in the modelling approach, one of the most robust SNPs was rs10477683 in the fibrillin 2 gene encoding fibrillin 2, a component of connective tissue microfibrils involved in elastic fiber assembly.	P/H (Performance and Health) Healthcare
(57)	Spain	During a 6-week period in the middle of a 9-month competitive season in 2015	Experimental randomized controlled trial	14 professional male players	Players completed matches in different field sizes: SSG, LSG, MG, CT, and FM drills and a friendly match (FM) over a 6-week period in 2015 during a 9-month competitive season. Comparisons were made between training data and the first 32 min of the LSG and two competitive FM per player. Post-task visual analogue scales recorded player effort and 10Hz GPS devices captured all movement patterns from strolling to sprint running.	Football	The dataset's cut-off point-values from four training drills (SSG, LSG, MG, and CT) and FM were assessed using decision tree induction for every parameter combination. Distance covered during jogging (2.3–3.3 m/s; >436 m), number of decelerations (≤730.5) and accelerations (≤663), and maximum velocity reached (>5.48 m/s) characterized the physical demands during competition (FM) with great variability amongst training drills.	P/H (Performance and Health) Performance
(58)	Australia	Season 2019–20	Prospective Cohort Study	26 professional male players were assessed during a competitive Australian Football League (AFL) season	Eight in-season rounds gathered (CK) 24–36 h before and 34–40 h after match. An athlete-tracking device measured match load. Generalized estimating equations and random forest models were used to assess how match-load indices and pre-match (CK) explained post-match (CK). Eight in-season rounds gathered (CK) 24–36 h before and 34–40 h after match. An athlete-tracking device measured match load.	Australian Football	They were used generalised estimating equations and random forest models to determine how match-load indices and pre-match (CK) explained post-match (CK). Most strongly associated with post-match (CK) were the number of impacts >3 g (*P* = 0.004) and game time (*P* = 0.016). Random forest found deceleration, acceleration, hits >3 g, and sprint distance as the strongest predictors with much less mistakes (130 vs. 316 U·L). Pre-match (CK) was 11% of post-match (CK), and data variability was considerable.	P/H (Performance and Health) Healthcare
(59)	Spain	2013/2014 season	Prospective observational cohort study	96 male professional soccer players. Player recruitment was conducted from 4 teams in the 1st (*n* = 25) and 2nd B (3 teams, *n* = 73) Spanish National Soccer League divisions.	The second week of August until the second week of May 2013/2014 HSIs of all players were prospectively collected. Before the season, players underwent psychological, neuromuscular, and personal testing for sport-related injury risk. Mid-season pre-season tests for each soccer club (end of July or beginning of August).	Football	There were 18 HSIs. Injury distribution was 55.6% dominant leg and 44.4% nondominant leg. The SmooteBoostM1 model with a cost-sensitive ADTree base classifier had the best assessment criteria (area under the receiver operating characteristic curve score = 0.837, true positive rate = 77.8%, true negative rate = 83.8%) and was the best at predicting HS.	P/H (Performance and Health) Healthcare
(60)	Belgium	During the 2015–2016 season, both pre-season and in-season	Prospective observational cohort study	26 professional male soccer players (mean ± SD age: 23.2 ± 3.7 years, weight: 77.5 ± 7.4 kg, height: 1.82 ± 0.06 m, body fat: 10.4% ± 1.9%) competing for the same team at the highest level in the Netherlands	Individual wellness predictions (fatigue, sleep quality, general muscle soreness, stress levels, and mood) were based on EL and IL indicators and presession wellness. EL and IL were calculated acutely and cumulatively. The absolute prediction error and effect size of the GBRT model's future wellness prediction were compared to the naive baseline.	Football	The GBRT model outperformed the baseline for the wellness items such as fatigue, general muscle soreness, stress levels, and mood. In addition, only the combination of EL, IL, and presession perceived wellness resulted in nontrivial effects for predicting future wellness. Including the cumulative load did not improve the predictive performances.	P/H (Performance and Health) Performance
(61)	Italy	Season 2015/2016	Prospective observational cohort study	22 elite soccer players (age = 21.96 ± 4.53 years; height = 180.68 cm ± 5.23 cm; weight = 72.36 ± 4.19 kg) competing in an Italian championship during the 2016/2017 season were recruited in this study	Although the rate of perceived exertion (RPE), training load (S-RPE), and global position system (GPS) are standard methodologies used in team sports to assess the internal and external workload; how the external workload affects RPE and S-RPE remains still unclear	Football	Machine learning applied to training workloads performed in the previous week has a strong effect on perceived exertion and training load. On the other hand, the analysis of our predictions shows higher accuracy for medium RPE and S-RPE values compared with the extremes	P/H (Performance and Health) Performance
(62)	Australia	19 rounds;12 indoor matches and 7 outdoor matches (*n* = 19) during the 2017	Prospective observational cohort study	37 male Australian football players (age: 23 ± 4 years, height: 187 cm ± 8 cm, mass: 86 ± 9 kg).	A change point quotient of between 1 and 15 was used. For these quotients, descriptive statistics, spectral features and a sum of skilled involvements were extracted	Australian Football	The strongest classification features in the model were spectral entropy and skewness. Offensive and defensive involvements were the weakest features for classification, suggesting skilled output is dependent on match circumstances. The methodology presented may have application in comparing the specificity of training to matches and designing match rotation strategies.	P/H (Performance and Health) Performance
(63)	Australia	2017 and 2018 Australian Football League Women's (AFLW) seasons	Retrospective observational cohort analysis.	Female Australian Football players	Thirteen performance metrics were collected from 56 AFLW matches in 2017 and 2018. Each athlete had absolute and relative values for 13 performance metrics in each quarter of matches. Eleven more features were recovered for each performance indicator, totalling 169. GEE and regression decision trees were run on different feature sets and dependent variables to create 22 models.	Australian Football	Compared to regression decision tree models, GEE had somewhat lower mean absolute errors across all dependent variables and feature sets. Total points scored described quarter outcome better than quarter score margin. The models’ strongest features were team differential and athlete 75th percentile inside 50 s.	P/H (Performance and Health) Performance
(64)	Spain	The study was conducted at the end of the pre-season phase in 2015 and 2016 (September).	Cross-sectional study	44 elite male futsal players from four different teams [16 players from a club engaged in the First (top) National Spanish Futsal division and 28 players from three clubs engaged in the Second National Futsal division]	44 elite male futsal players were examined for dynamic postural control (y-balance), isokinetic (concentric and eccentric) knee flexion and extension, isometric hip abduction and adduction, lower limb joint ROM, and core stability Features were selected before Tabu search generated leg BNs. The BN models updated beliefs to explore neuromuscular performance parameters’ influence on dynamic postural control individually and simultaneously.	Football Indoor	The dominant (AUC = 0.899) and non-dominant (AUC = 0.879) legs of the BNs created utilising the selected features by correlation attribute evaluator and chi squared had the highest evaluation criteria (AUC).	P/H (Performance and Health) Performance

### Studies characteristics

**Figure 3:**
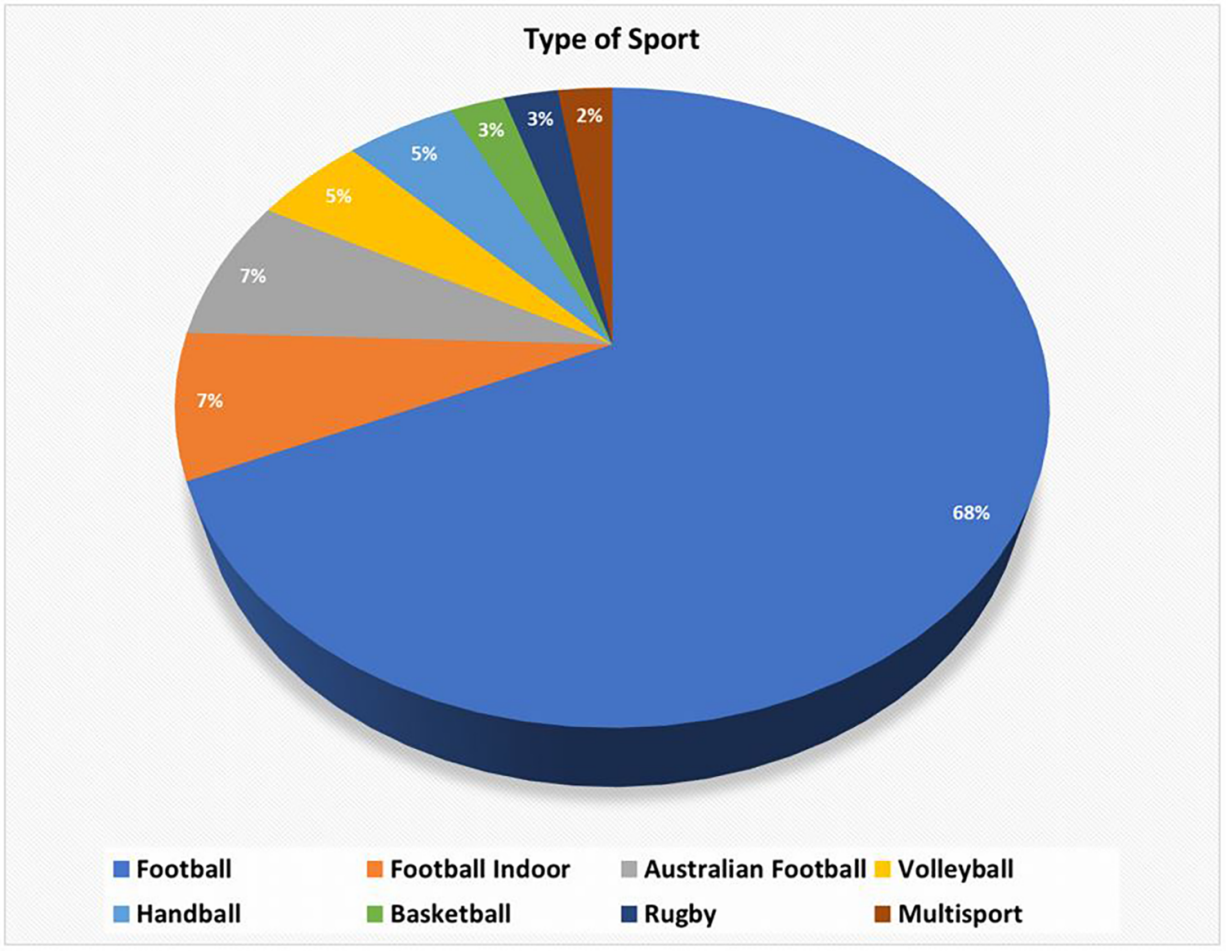
Distribution of articles by type of sport.

Of the 32 studies identified, the majority are on football (soccer) (67%) (Figure 3) which might be justified by the fact that it is the most developed area of sport and business and where the most resources are invested to improve performance and achieve results.

A population of 2,823 professional athletes were examined in the 32 papers that were screened, of which 1,845 (or 65.36%) were men and 978 (34.64%) were women. Thus, the actual distribution of papers is as follows: 26 pertain to studies on men and 3 to studies on women, with two papers containing a mixed population. Lastly, one study was identified as subject-free and match-centered, and it was not accounted for.

The sports levels seen are those filtered by the PRISMA-ScR search, and they are adults from high-level senior teams who develop in professional leagues.

An analysis of the application domains within the research teams reveals that the majority of studies focus on enhancing healthcare (*n* = 17) through the prevention of injuries and, secondarily, performance (*n* = 15) improvement. In this regard, AI is used or sought to assist in this endeavour.

### Main artificial intelligence (AI) technique or method in team sports

**Figure 4:**
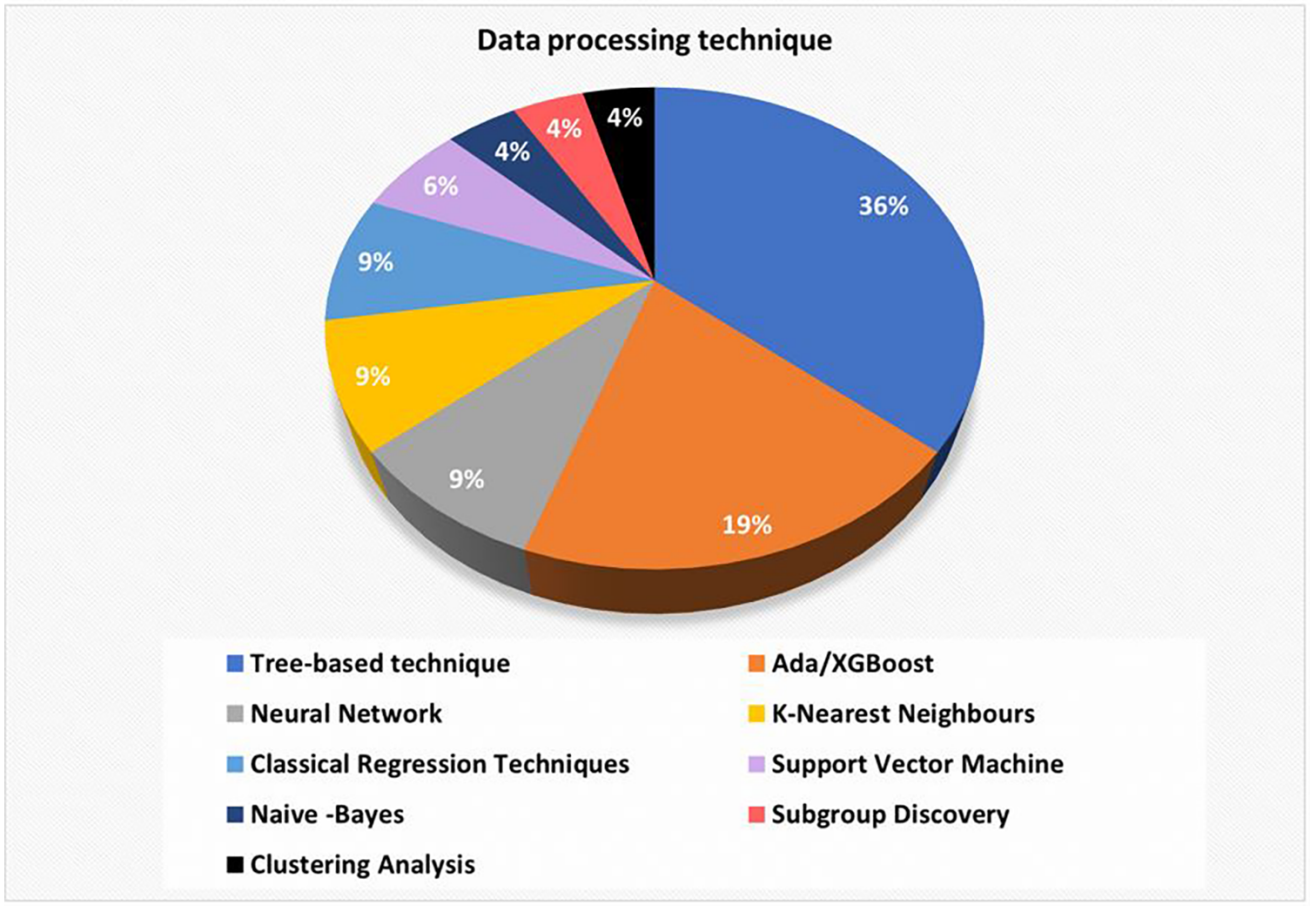
Distribution of articles by data processing technique.

In the 32 selected articles, it can be observed that the most frequently used AI-based and non-AI-methods for data processing are (Figure 4): Tree-based techniques (36%), Ada/XGBoost (19%), Neural Networks (9%), K-Nearest Neighbours (9%) Classical Regression Techniques (9%) and Support Vector Machines (6%).

It should be noted that AI techniques are normally combined with classical statistical methods to complement and help reveal information due to the complexity of the relationships sought in the studies.

The synthesis of results (Table 4), has been evaluated and showed the diverse methodologies utilized to assess each type of artificial intelligence applied, including the Gradient Boosted Regression Trees (GBRT) model, the Area Under the Receiver Operating Characteristic (AUC-ROC) curve, Key Performance Indicators (KPI), Uniform Manifold Approximation and Projection (UMAP), Kernel Principal Component Analysis (k-PCA), t-Distributed Stochastic Neighbor Embedding (t-SNE), Ordinary Least Squares (OLS) regression, Root Mean Square Error (RMSE), Alternating Decision Trees (ADTree), and Decision Trees (DT).

This comprehensive evaluation is critical in understanding the strengths and limitations of each AI methodology within the context of sports performance and healthcare. By scrutinizing how each AI technique contributes to the analysis and prediction of athletic performance and injury prevention, we can discern the most effective tools for specific applications.

For instance, the GBRT model, known for its predictive accuracy in regression and classification problems, is assessed for its efficacy in predicting injury risk and performance outcomes (60). The AUC-ROC curve, a measure of the ability of a classifier to distinguish between classes, is used to evaluate the performance of predictive models in injury severity and return-to-play predictions (45, 52, 64).

KPIs, on the other hand, are analyzed for their role in quantifying physical demands and wellness metrics, providing a tangible measure of athlete performance and health. Dimensionality reduction techniques such as UMAP, k-PCA, and t-SNE are evaluated for their effectiveness in visualizing high-dimensional data, aiding in the identification of patterns and relationships that are not immediately apparent (36).

OLS regression and RMSE are scrutinized for their application in predictive modeling, assessing their accuracy in forecasting outcomes based on linear relationships (43). Lastly, the ADTree and DT methodologies are evaluated for their utility in classifying and predicting outcomes based on hierarchical decision-making processes (52, 64).

By valuing the different methodologies for assessing each type of artificial intelligence applied, our synthesis of results not only highlights the versatility and potential of AI in sports science but also guides future research and application towards the most effective and efficient tools for enhancing athletic performance and healthcare.

### Research topics

When reviewing the articles selected for our study, it became evident that the research topics could be broadly categorized into two principal domains: Performance and Healthcare. This bifurcation not only aids in a structured analysis but also aligns with the overarching aim of enhancing athletic performance while ensuring the well-being of the athletes.

In the domain of Performance, our investigation revealed intriguing insights into the utilization of GPS data for tracking, profiling, and decision-making purposes. The ability to monitor athletes' movements in real-time has revolutionized the way performance is analyzed, offering a granular view of their capabilities and areas for improvement. Furthermore, the aspect of Wellness Control emerged as a critical factor. By monitoring athletes' wellness, we can discern its direct impact on performance levels. Here, AI-driven insights have provided new lens through which wellness can be quantified and optimized.

Predicting future performance from anthropometric data and performance tests has shown promising results. This approach underscores the importance of individual physical characteristics and their correlation with performance outcomes. Additionally, the utilization of Key Performance Indicators (KPIs) through AI has provided a nuanced understanding of the physical demands placed on athletes during training and matches. This knowledge is pivotal in tailoring training programs that maximize performance while minimizing the risk of injury.

Turning our attention to Healthcare, the research delved into Injury Prevention and Prediction. Through a multifactorial analysis, we have begun to unravel the complex web of factors contributing to injury risk among elite football players. Predictive modeling has further enhanced our ability to forecast injury severity, classify muscle injuries, and predict return-to-play timelines post-hamstring injuries with greater accuracy.

The correlation between External Load Data and injury risk, along with fatigue assessment, has shed light on the delicate balance between training load and athlete well-being. By monitoring external load, we can better understand its relationship with injury risk and athlete fatigue, aiding in the development of more informed training and recovery protocols.

Moreover, the integration of Psychological and Physiological Factors for injury risk screening has highlighted the multifaceted nature of injuries. Recognizing that both mental and physical states play a critical role in the occurrence of injuries has led to more holistic approaches to injury prevention. Lastly, the innovative integration of Blood Biochemical Markers and Genetics for personalized injury risk assessment marks a significant leap towards customized healthcare in sports. This approach enables a deeper understanding of each athlete's unique physiological makeup, paving the way for personalized injury prevention strategies.

In summary, the thematic analysis of the selected articles has not only enriched our understanding of the intricacies involved in sports performance and healthcare but also highlighted the potential of technology and data analytics in pushing the boundaries of what is possible in sports science.

## Discussion

The primary objective of this research is to provide a global perspective on the state of the art in terms of the use of artificial intelligence in various areas of advisory services. These aspects include topics such as sports performance and healthcare. As a brief methodology recap, it has to be emphasized how the thematic analysis led to the identification of the discussed topics.

Some authors have previously described how team sports can benefit from these automated or artificial intelligence technologies (28). Historically, research has been carried out employing a scientific framework and methodology founded on statistical investigations. Nevertheless, these foundational statistical studies are being progressively helped and superseded by more intricate models and methodologies derived from artificial intelligence. This could improve the precision with which these expert models or their outputs may be implemented in the actual world. AI is normally integrated with statistical methods to enhance information, injury prediction, and player performance monitoring.

Given the current state of the sports framework, the complexity of the data processing methods involved, and the intrinsic properties of the data, it is critical to form horizontal and multidisciplinary teams to maximise the potential of artificial intelligence and machine learning technologies.

One area of investigation that could not be detected with our search strings is cardiological tests such as electrocardiograms utilised in team sports, probably due to the variations in the papers' formulations. Certainly, some authors have studied the gaps and exerted considerable effort in this domain (65).

### Performance

There are numerous references in which AI has been applied to this facet of sports. An abundance of data has been collected since the implementation of tracking technologies like the Global Positioning System (GPS) or Global Navigation Satellite System (GNSS). Additional functionalities have been incorporated into the systems to monitor distances, speeds, decelerations, and accelerations; this information is updated regularly and throughout contests and training.

#### GPS data tracking, profiling and decision-making

GPS devices are extremely useful since they enable the investigation of a vast array of physical concepts in order to precisely define what, when, and where it occurs during matches and training. Certain authors employed this methodology to assess player performance during training and matches within the setting of Serie A. Their research utilised machine learning models such as XGBoost and Decision Trees to illustrate the progressive improvement in the accuracy of XGBoost as the season progressed. By employing real-world scenarios and cross-validation techniques, they gained valuable insights into the dynamics of performance (47). Similarly, additional researchers conducted a study of clustering and performance profiling utilising GPS data and 38 male football players, respectively, in order to identify unique performance profiles. The incorporation of machine learning techniques in this study enhances comprehension of the diverse performance attributes exhibited by players, hence facilitating the development of customised training and optimization approaches (48). In an effort to forecast individual acceleration-velocity profiles in real-world scenarios utilising GNSS readings, some researchers sought to further the individualization of acceleration-velocity profiling in the context of professional male footballers. The development of multivariate models, which incorporated long short-term memory neural networks and time series forecasting, highlighted the capacity of machine learning to provide individualised performance insights. A model description and multivariate approaches were built with the following techniques in mind: time series forecasting, ridge regularisation, and long short-term memory neural networks (46). Finally, match performance prediction and substitution decision support are critical in team sports. To this end, some authors applied machine learning models such as Random Forest and Decision Trees to in-match position tracking data from 302 competitive professional soccer matches to forecast player performance levels. The research, which utilised data acquired retrospectively, illustrated the capability of early match forecasts to assist coaches in making well-informed substitute judgments (53).

#### Wellness related to load control

Developing prediction models for the wellbeing of elite football players was a subject that was expounded upon by numerous authors. An association between well-being indicators and training load was determined by analysing 28 sub-elite football players over the course of the 2017–2018 season. By utilising a machine learning methodology, they successfully predicted wellbeing markers such as the training load completed the day before with a noteworthy ordinal regression accuracy of 39% (2%). This demonstrates how machine learning has the ability to predict the well-being of football players (51). In volleyball, some authors used XGBoost, random forest regression, and subgroup discovery to study male national volleyball teams' well-being, RPE, and readiness using questionnaires. The study examines defensive and attacking game phases and shows how machine learning can analyses volleyball performance patterns (44).

Professional football players' fatigue prediction utilising Random Forest, Lightgbm, and gradient Boosting Regressor machine learning models is intriguing. FatigueNet accurately predicted Rating of Perceived Exertion (RPE), demonstrating the importance of machine learning in player tiredness prediction (49). Other writers used FatigueNet and stressed the importance of perceived exertion rating in prediction. Machine learning algorithms like Random Forest and the “FatigueNet” model were used to study movement characteristics and well-being. They stressed the efficacy of machine learning in predicting well-being, providing valuable insights for player management and performance improvement (50). Gradient-boosted regression trees (GBRT) were used to create predictive models for individual wellness variables in 26 professional male football players for RPE prediction. The GBRT model outperformed baseline techniques in predicting future wellbeing using acute and cumulative load indicators (60). Following the 2015/2016 study of professional soccer players' RPE and training load, this study examined the effects of training workloads on perceived exertion and training load. The research used RPE, S-RPE, and GPS to show that the previous week workloads strongly affected subsequent perceived effort and training load. The study emphasises machine learning's usefulness in understanding the complex links between external tasks and subjective player experiences (61).

In summary, the use of AI and ML in football performance modelling has changed sports performance research. Multiple studies highlight the adaptability of these technologies, from forecasting sub-elite football players' well-being to studying soccer training practise effects. These improvements affect volleyball, handball, and football.

#### Predicting performance from anthropometric and testing

Other authors have used radial-basis function neural networks to predict athletic performance in women's handball using anthropometric metrics, power tests, and jump tests from 59 female players. A total of 23 anthropometric parameters, power tests (Wingate), and jump tests (CMJs) were combined. The study demonstrates the potential of machine learning to predict handball players' performance, with *R*^2^ scores ranging from 0.86 to 0.97 (55). In a study of elite male futsal players, Bayesian networks were used to explore how neuromuscular performance affects dynamic postural control. The study showed that Bayesian networks using the Tabu search algorithm may capture the interaction of performance characteristics, providing futsal players with significant insights. Dynamic postural control (y-balance), isokinetic (concentric and eccentric) knee flexion and extension, isometric hip abduction and adduction, lower limb joint ROM, and core stability were measured as the features were chosen. The dominant (AUC = 0.899) and non-dominant (AUC = 0.879) legs of the BNs generated using correlation attribute evaluator and *χ*^2^ had the highest assessment requirements (AUC) (64).

#### Key performance indicators (KPI): AI helps to understand physical demands on training and matches

In football, some authors used experimental randomised controlled trials to test the effects of various training drills on soccer performance during matches in different field sizes. They used decision tree induction to determine cut-off point values for different parameters. The research uses machine learning to understand drills' physical demands and develop customised training methods (57). In order to improve KPI in Australian football, some authors have developed spectral features and classification using Random Forest Models to evaluate segment similarity and classify spectral characteristics. The 37 Australian football players study showed that spectral entropy and skewness are important in distinguishing skilled output and that machine learning may be used to tailor training specificity to conditions. Offensive and defensive involvements were the lowest categorization features, suggesting match conditions affect skilful performance. The methodology may be used to compare training specificity to matches and build match rotation methods (62). Finally, additional authors used generalised estimating equations (GEE) and regression decision trees to evaluate performance indicators from the Australian Football League Women's (AFLW) seasons to reveal crucial aspects not found in conventional statistics. The study showed that team differentials and athlete percentiles can describe quarter outcomes, demonstrating the power of machine learning to analyse performance (63).

### Healthcare

Sport is increasingly emphasising health, which is more than just the absence of disease or injury. Anyway, most AI-based solution research focuses on harm prevention.

#### Injury prevention and prediction

The majority of studies were focused on predicting non-contact lower-body injuries in male professional players. One of them employed three decision-making methods, with the XGBoost algorithm showing the most promising results, achieving high precision, recall, and F1-score (35). Blood samples were utilised to customise machine learning (ML) models for injury prediction in an intriguing study involving eighteen male professional soccer players. By including blood parameters and GPS-measured exterior workloads, the research study achieved an accuracy of 63%, outperforming models that exclusively relied on the features of the training workloads (33). An additional body of research investigated the prediction of injuries among professional football players, with a particular emphasis on the implementation of screening data within a gradient-boosted model. Incorporating 112 adult male football players, the research exhibited encouraging cross-validated performance and proposed applicability to novel situations through the implementation of gradient boosting with ROSE upsampling in a leave-one-out cross-validation scheme for data processing (34).

Other writers used dimension reduction to assess elite female soccer players' injury signs. They compared UMAP (Uniform Manifold Approximation and Projection) to non-linear kernel principal component analysis (k-PCA) and t-distributed stochastic neighbour embedding for damage pattern detection (t-SNE). UMAP identified damage indicators using grid search, suggesting it may be better in this situation (36).

In an interesting and broad analysis of 791 female elite handball and soccer players from 2007 to 2015, researchers investigated different machine learning algorithms for predicting anterior cruciate ligament (ACL) injuries. Using a robust technique that addressed chance and random changes, the study indicated that the ideal linear support vector machine classifier had a mean AUC-ROC of 0.63. However, AUC-ROC values varied from 0.51 to 0.69 among approaches and repetitions. Addressing class disparities did not improve prediction outcomes (45).

In tandem with the preceding authors and imbalance detection, other research examines muscular strength metrics to predict injuries in professional male football players. Preseason study uses subgroup identification technique to assess 77 athletes, 92 injury cases and 186 healthy cases. Countermovement leap, eccentric hamstring strength, and isometric hip adductor and abductor strength are examined. Subgroup Discovery data mining works well with tiny datasets and reveals patterns. The study found a higher injury risk for between-limb abduction imbalance three weeks before to the occurrence, exceeding a threshold of ≥0.97. In addition, a right leg adduction muscle strength threshold of ≤1.01 is a significant risk factor one week prior to injury. These findings demonstrate the importance of monitoring key strength metrics and their complex interaction in injury prediction, providing practical insights for professional football injury prevention strategies (38).

One study conducted a Subgroup Discovery machine learning analysis of 14 elite male volleyball players' injuries, illness, and perceived wellness during 24 weeks of the 2018 international season. The study monitors 1,112 professional player questionnaires from training and matches. Based on degree of complaints, injuries, or pain and their impact on performance and training volume, players are divided into three groups (Q1–Q3). The results shed light on how physical well-being, training load, and elite volleyball performance interact. This complete assessment helps elite male volleyball players optimise training and injury avoidance (41).

##### Multi-factorial analysis for predicting injury risk in elite football players

One study analyses 36 male elite football players and 22 independent variables, including player information, body composition, physical fitness, and season injuries, to predict injury risk. Net elastic analysis is pursued using traditional regression models (OLS), shrinkage regression, and stepwise regression. The data show that defensive and forward sectorial positions, body height, sit-and-reach performance, 1-min push-ups, handgrip strength, and 35 m linear speed affect injury risk. The investigation showed that the most accurate predictive model predicts elite footballer injury risk with a root mean square error (RMSE) of 0.591. This holistic approach illuminates the multifaceted nature of injury vulnerability, aiding injury prevention and athlete well-being (43)**.**

##### Predictive modelling of soccer player injury severity

In a remarkable study spanning the 2013/2014 season, some authors (59) conducted a comprehensive analysis of injury prediction methodologies in male professional football players. The cost-sensitive ADTree base classifier-based SmooteBoostM1 model was added in preseason tests. This model performed well with an area under the receiver operating characteristic curve score of 0.837, a true positive rate of 77.8%, and a true negative rate of 83.8 percent. The meticulous investigation of 18 injuries showed 55.6 percent in the dominant limb and 44.4 percent in the nondominant leg. This study sheds light on predictive modelling for injury risk assessment and the distribution and features of injuries among male professional football players during the season.

##### Muscle injury classification and expert predictions

Utilizing the British Athletic Muscle Injury Classification (BAMIC), researchers estimated muscle injuries among male football players. It was shown that XGBoost performed the best among the models that had their performance enhanced by including expert forecasts (40).

##### Predictive analysis of return-to-play in hamstring injuries

Hamstring injuries in male professional football players from February 2010 to February 2020 were examined in this retrospective observational cohort study. The study uses MLG-R, a comprehensive classification system, and linear regression, random forest, and eXtreme Gradient Boosting to assess return-to-play factors for 76 injuries involving 42 players. 65.8% of injuries were grade 3rd, with the biceps femoris long head frequently injured. MLG-R shows strong predictive power with a mean absolute error of 9.8 days and an *R*^2^ of 0.48. Location and grade of the injury at the free tendon of the biceps femoris long head determine return-to-play. The study confirms the MLG-R classification system's accuracy in predicting elite footballers' hamstring injury return-to-play times, providing valuable injury management and prevention insights (42).

### External load data, injury risk and fatigue assessment

Some authors observed elite soccer players and compared machine learning models to predict high chronic exertional compartment (HCE) severity. The study highlighted the importance of injury mechanisms and pre-injury incidents, linking them with severity (37). Others assessed professional male football players using a subgroup discovery algorithm. The study revealed that injuries were more likely when there was an imbalance in abduction strength or right leg adduction muscular strength remained unchanged or deteriorated (38). A Research monitored elite soccer players, employing machine learning techniques like Extreme Gradient Boosting (XGBoost) and Random Forest Regression to analyze external load data. Random Forest Regression provided the best performance in assessing fatigue or neuromuscular readiness (39).

We can also develop models that enable us to comprehend soccer player injuries based on anthropometric or screening differences (43), as well as the control of the internal and external load in soccer (54) or in Australian Football Indeed in this last discipline some authors claim to have made contributions to injuries and their relationship with specific actions like deceleration and acceleration or impacts (58).

An intriguing study that integrated external and internal strain in professional soccer comprised 40 male players from a top French Ligue 2 club from June 2017 to May 2018. We compared non-linear models, specifically interpretable tree-based classification machine-learning methods. Understanding injury risk from internal and exterior load features required these algorithms. Evaluations included algorithm accuracy, precision, recall, and receiver operator characteristic curve area. The study found that training burden, perceptive well-being, and machine learning avoid top soccer injuries (54).

Another study uses a predictive approach to examine the complex relationship between external load variables and professional soccer players' RPE ratings. The study examines 58 GPS external load factors and 30 accelerometer variables using linear regression, K-NN, decision trees, random forest, elastic net regression, and XGBoost. These factors and RPE-derived internal loads are studied throughout 151 training sessions and 44 matches in a season. The machine learning models predict high chronic external load (HCE) severity with an average area under the receiver operating characteristic curve of 0.73 for male and 0.70 for mixed datasets. Injury mechanisms like “head-to-head” and “knee-to-head,” as well as match events like “corner kicks” and “throw-ins,” are good predictors. This study highlights the importance of specific actions and their correlation with soccer player injury severity, advancing injury prediction (37).

### Integrating psychological and physiological factors for injury risk screening

This study incorporates psychological and physiological aspects to create injury risk screening models for male and female indoor football players. Decision trees with C4.5 and ADTree, Support Vector Machines with SMO, and k-Nearest Neighbor were used in the analysis of 139 participants (KNN). The models used psychological risk variables, self-perceived chronic ankle instability, neuromuscular risk factors, and injury monitoring data to optimise each base classifier using MultiSearch. The screening models, based on range of motion (ROM) and dynamic postural control features, had moderate accuracy (AUC scores of 0.701–0.767). The “model of best fit,” with two hip and two ankle ROM values and 10 classifiers, had an AUC of 0.767, 85 percent true positive, and 62% true negative. This complex approach emphasises the importance of psychological and physiological factors in indoor football injury risk assessment (52)**.**

#### Integrating blood biochemical markers and genetic for personalized injury risk assessment

A pioneering study examined the complex link between blood factors and athlete injury risk assessment. The study carefully examined red cell data, ferritin, testosterone, and cortisol to build player profiles. A complete method using internal and exterior load data was recommended by the study. The combination of Decision Trees (DT), Dummy, and XGBoost (XGB) algorithms improved injury risk prediction. This novel approach shows how physiological markers can improve injury risk assessments, enabling more effective sports injury prevention strategies (33).

In a detailed study of male Australian football, twenty-six professional players were studied during one AFL season. The study used parametric and machine-learning analysis to reveal the complex relationship between physical load indices and muscle injury during intensive competition. The study found impacts exceeding 3 g and high-intensity running variables as robust predictors of muscle injury, as indicated by generalised estimating equations and random forest models. Athlete tracking data like deceleration, acceleration, impacts >3 g, and sprint distance were better predictors than traditional approaches. Impacts >3 g and game time were best predictors of post-match creatine kinase (CK) levels. Intriguingly, pre-match (CK) only made up 11% of post-match (CK), showing data unpredictability. This study illuminates muscle injury patterns during AFL competition and emphasises the need of tracking measurements in forecasting and analysing post-match (CK) levels (58).

Using genetics, a 2008–2018 case-control study examined 363 elite soccer, futsal, basketball, handball, and roller hockey players from FC Barcelona, Spain. The study investigated the complex relationship between genetics and tendinopathy in high-performance athletes. A machine learning-based multivariate modelling technique using support vector machine and random forest algorithms was used to analyse the influence of SNPs on tendinopathy susceptibility. The study began with a hypothesis-free genome-wide association study of 495,837 SNPs, then targeted examination of 58 SNPs identified as risk factors in the literature. The predictive model included robust SNPs after synthetic variant imputation, with fibrillin 2 gene rs10477683 being influential. In the multidimensional modelling technique, fibrillin 2, a key component of connective tissue microfibrils involved in elastic fibre construction, showed significant connections. These findings illuminate the genetics of elite athlete tendinopathy, enabling tailored injury preventive measures (56).

## Limitations

During our study, we ran into a few key limitations. Firstly, we focused mainly on strength and conditioning when talking about “performance,” leaving out areas like technical skills, tactics, and talent spotting. We used the STROBE tool to help find and fix weaknesses in the studies we looked at, making our review stronger.

One big issue was how we set up our search, which missed some important health studies, especially those on heart health. This mistake shows we need a wider search strategy in the future to cover more health topics. We also didn't include enough about collective team sports like baseball, cricket, hockey or others, and we completely missed sports for para-athletes. This shows the need for future studies to cover a broader range of sports.

Another problem was that the studies we looked at didn't share their data publicly, making it hard to check their findings. This points to a need for more openness in sharing data for scientific research.

We also noticed not enough research on professional women's sports, which is a gap that needs filling. This lack of information is a missed opportunity to better understand and improve performance in a wider range of sports.

## Conclusion

In conclusion, after conducting an extensive review and scoping analysis of the pertinent literature, we have determined that artificial intelligence and machine learning have the potential to bring about significant changes in numerous aspects of team sports participation and coaching, keeping track of the health and safety of individuals and teams, including injuries. Further clinical investigations utilising rigorous methodologies are required to extract more dependable conclusions from the vast amount of data that the teams are generating. By confronting the limitations of current research and embracing a more inclusive and comprehensive methodology, the field of sports science can continue to evolve, offering valuable insights and practical applications that enhance athletic performance and healthcare across the sporting spectrum.

### Practical applications and future prospects

The issues we found point out where sports science can improve. By tackling these problems, future research can give us a fuller picture of athletic performance and health, helping athletes in many different sports.

We suggest making training programs that meet the specific needs of different sports, including those for para-athletes and women's teams. Also, sharing data more openly can speed up progress in sports science by making studies easier to verify.

Looking ahead, research should look at athletic performance in a more general sense, including technical, tactical, and talent aspects. Expanding studies to more sports and including para-athletic activities will give us a better understanding of performance. Finally, focusing more on women's sports is crucial to make sure research benefits all athletes equally.

## Footnotes

^1^https://github.com/dradolfomunoz/AIEliteSportsTeamPerformance/blob/main/Study_Protocol.
